# Biomechanical effects of soft and rigid passive back occupational exoskeletons during load-carrying and static trunk bending tasks in the aeronautics industry

**DOI:** 10.1017/wtc.2025.10029

**Published:** 2025-10-03

**Authors:** Thomas Albouy, Guillaume Mornieux, Estelle Chin, Mohsen Zare

**Affiliations:** 1 DevAH, Université de Lorraine, 54000 Nancy, France; 2 Safran Landing Systems, 64400 Bidos, France; 3 ELLIADD (UR4661), UTBM, Université Marie et Louis Pasteur, 25200 Montbéliard, France

**Keywords:** Biomechanics, Exoskeletons, Exosuits, Industry, Sensors

## Abstract

The manufacturing industry, notably the aeronautics sector, involves tasks presenting risks of low back pain. One of the preventive strategies could be the use of passive back exoskeletons, which have demonstrated benefits during activities involving trunk bending. This study aims to evaluate the effects of four passive back exoskeletons on trunk neuromuscular activity, kinematics, and perceived discomfort during polishing tasks simulated in a laboratory setting. Nineteen participants performed four tasks (two static bending tasks and two load-carrying tasks) without and with two soft (CORFOR and BionicBack) and two rigid (BackX and Laevo FLEX) exoskeletons. The results showed varying effects depending on the tested exoskeleton model, beyond the distinction between rigid and soft designs. Reductions in lumbar erector spinae (LES) neuromuscular activity were observed with Laevo FLEX and CORFOR during static tasks compared to the condition without exoskeleton (8–18%; *p* < .05). However, reductions in LES muscle activity were not significant during load carrying. Biceps femoris neuromuscular activity was significantly lower in the four tasks when using the Laevo FLEX, with reductions ranging from 8 to 17% (*p* < .01). The two rigid exoskeletons decreased perceived back discomfort across all tasks (*p* < .05). Finally, the BionicBack exoskeleton significantly altered participants’ kinematics across all four tasks, reducing both trunk range of motion and average flexion (p < .05). The Laevo FLEX exoskeleton was the only one to significantly reduce both neuromuscular activity and perceived back discomfort, while causing no adverse effects, appearing advantageous when polishing in the aeronautical industry.

## 1. Introduction

### 1.1. Background and motivation

In the aeronautics industry, tasks involving material handling, maintenance, and repair present high risks for musculoskeletal injuries, particularly low back pain (LBP; Asadi et al., 2019). The development of LBP is associated with work-related risk factors, including carrying loads and sustained awkward postures such as trunk flexion (Costa da and Vieira, 2010). Studies in airline maintenance confirm that LBP is the most affected body region, with 12-month prevalence rates of 30–45% and 7-day rates of 16–27% (Menegon and Fischer, 2012; Nogueira et al., 2012). Additionally, 57% of observed tasks across aeronautics work are classified as high-risk for musculoskeletal strain (Asadi et al., 2019). Beyond its impact on workers’ quality of life, LBP is a leading cause of absenteeism and lost workplace productivity, affecting industrial performance (Fan and Straube, 2016; EU-OSHA, 2019).

Finding solutions to reduce physical workload in tasks that require high levels of observation, flexibility, and decision-making, such as those in the aeronautic industry, remains a significant challenge. Furthermore, preventive measures are sometimes ineffective, as certain tasks are inherently difficult to modify due to their complexity and operational constraints (De Looze et al., 2016; Govaerts et al., 2024). One of the preventive strategies to deal with LBP risks that has gained increasing attention with recent technological advancements is the use of passive exoskeletons. These non-powered assistive devices are generally classified into soft and rigid designs. Soft exoskeletons are lightweight, flexible, and composed of elastic materials and straps. In contrast, rigid exoskeletons incorporate metal or composite structural frames and spring mechanisms or torsion elements that act as hip and trunk extensors (Toxiri et al., 2019; Schwartz et al., 2021; Refai et al., 2024). Passive back exoskeletons, therefore, aim to store and release energy during bending and lifting, and have shown potential benefits in both dynamic lifting activities and prolonged static posture (De Looze et al., 2016; Theurel and Desbrosses, 2019; Kermavnar et al., 2021).

A systematic review by Kermavnar et al. (2021) reported that passive back-support exoskeletons reduced neuromuscular activity in the erector spinae (ES) by ~18% (range: 6–35%) during lifting and 36% (range: 14–61%) during static bending. They also reported reductions in hip extensor muscle activity, particularly in the biceps femoris (BF), by ~16% during lifting and 22% during static bending.

Regarding rigid exoskeletons, the BackX exoskeleton has been shown to reduce back muscle activity by up to 47% (Madinei et al., 2020b) during trunk bending tasks and by 13–22% during load-carrying tasks (Poon et al., 2019; Alemi et al., 2020; Madinei et al., 2020a). The Laevo FLEX or its earlier models demonstrate a reduction in back muscle activity ranging from 11 to 57% during trunk bending tasks (Bosch et al., 2016; Madinei et al., 2020b; Refai et al., 2024) and between 12 and 16% during load carrying (Alemi et al., 2020; Luger et al., 2021).

Few soft exoskeletons have been developed and studied to date (Toxiri et al., 2019; Kermavnar et al., 2021; Schwartz et al., 2021), but still show promising results, with reductions in muscle activity of 7–43% (Lamers et al., 2018;Schwartz et al., 2021; Thevenot et al., 2025). During load carrying, earlier versions of the CORFOR exoskeleton reduced muscle activity by about 7% (Schwartz et al., 2021; Thevenot et al., 2025) and BionicBack reduced it by about 20% during the lifting and the lowering phases (Reimeir et al., 2023).

### 1.2. Current limitations and research gap

The benefits of back exoskeletons vary across studies, largely due to differences in task characteristics (e.g., static vs. dynamic, trunk flexion angles, loads lifted) and exoskeleton features (Theurel and Desbrosses, 2019; Schwartz et al., 2022), notably soft or rigid. Furthermore, negative effects may appear, like discomfort (Bosch et al., 2016; Amandels et al., 2019; Alemi et al., 2020), overuse of antagonist muscles (Baltrusch et al., 2019; Koopman et al., 2019; Luger et al., 2021) or changes in the user’s kinematics (Bosch et al., 2016; Baltrusch et al., 2019; Luger et al., 2021).

Testing exoskeleton performance in conditions that closely replicate real-world scenarios is, therefore, crucial for understanding their impact and validating their suitability for the task. Theurel and Desbrosses (2019) emphasize the need to expand research beyond conventional trunk flexion/extension tasks, as previous studies do not fully capture the complexity and variability of real industrial work environments. Studies performed in real-world settings or under controlled, task-relevant conditions are necessary to gain deeper insights into the effects of exoskeletons on workers in the aeronautics sector.

To our knowledge, no study to date has investigated the impact of using a back exoskeleton during tasks in the aeronautics industry. To address this gap, this study aims to evaluate the biomechanical effects of four passive back exoskeletons (including soft- and rigid-based designs) during polishing tasks simulated in a laboratory setting. We hypothesized that exoskeletons would decrease ES neuromuscular activity, reduce perceived back discomfort, and not cause adverse effects (including muscle overuse, changes in trunk, hip, and knee kinematics, or increased discomfort).

## 2. Materials and methods

### 2.1. Participants

Nineteen male subjects (25 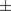 7 years, 180 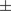 6 cm, and 76 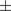 10 kg) were recruited for this study. This sample size was estimated based on a power analysis using the effect size reported for ES neuromuscular activity in response to the CORFOR exoskeleton use (Schwartz et al., 2021), that is, likely the least assistive device, for an alpha error level of 0.05 and a power of 80%. All participants engaged in moderate physical activity at least twice a week and had no prior or current musculoskeletal disorders. The study included only male participants to eliminate potential sex-related effects (Alemi et al., 2020; Schwartz et al., 2021) and to ensure a representative sample of the target population, which, in this specific aeronautics field, consists mainly of men. The participants were university students and staff, and therefore not aeronautics professionals. They were also not familiar with exoskeletons.

To minimize the risk of neuromuscular fatigue, participants were instructed to avoid strenuous physical activity for at least 48 hr before the experiment. Ethical approval was granted by the Research Ethics Committee (CERUBFC-2023-10-05-043) and all participants provided written informed consent before participation.

### 2.2. Exoskeletons

Four passive back exoskeletons (Table 1) were used in this study, with selection primarily guided by the literature reviewed above regarding their documented effects. Practical aspects such as price, mass, size, adjustability, and availability were also considered.

**Table 1.** Characteristics of the four exoskeletons tested in this study

Exoskeleton model	CORFOR V5	BionicBack	BackX	Laevo FLEX
Manufacturer	SIDECT	hTRIUS	SuitX, Ottobock	Laevo
Web link	www.corfor.fr	www.htrius.com	www.suitx.com	www.laevo-exoskeletons.com
Picture	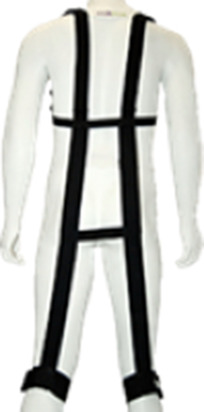	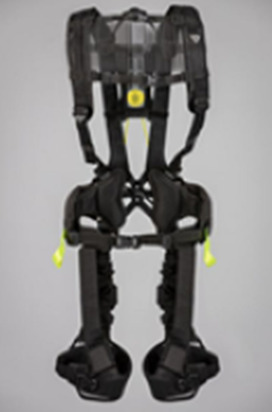	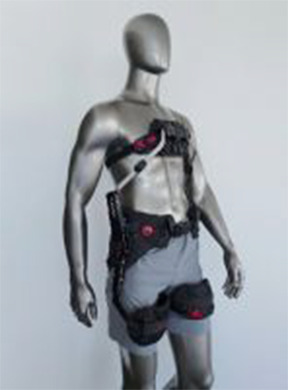	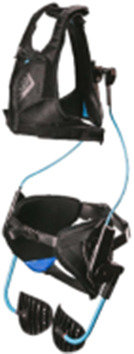
Type of mechanism	Passive Soft	Passive Soft, partially rigid	Passive Rigid	Passive Rigid
Functional mechanism	Elastic bands along the back	Elastic bands from a rigid structure to lower interfaces	Pivot at the hip, torque from a gas spring	Pivot at the hip, torque from a gas spring
Interfaces	- Shoulder straps - Straps below knees	- Shoulder straps - Thigh straps	- Chest pad - Curved pads at the thighs	- Textile vest - Curved pads at the thighs
Weight	160 g	1.18 kg	3.2 kg	2.5–4 kg
Possible disengagement	No	Yes, manual	Yes, manual	No real disengagement, but pads can be put on the side
Sizes/settings	8 sizes	One adjustable size	One adjustable size	One size with interchangeable parts
Price order	€135	€2,800	€5,900	€5,400

#### 2.2.1. Rigid exoskeletons

Two rigid passive exoskeletons were tested in this study: BackX (20ES90 = 1 model, SuitX by Ottobock, Germany) and Laevo FLEX (Laevo, The Netherlands). These devices provide torque support around their main joint, which is aligned with the trochanter major of the user. Support is delivered through thigh interfaces (curved pads) connected to a pivot mechanism at the hip, where torque is generated by a gas spring system. This torque is transmitted between the thighs and the upper body via a chest interface, enabling load support. The BackX transmits force to the upper body via a rigid chest pad, while the Laevo FLEX utilizes a textile vest.

#### 2.2.2. Soft exoskeletons

BionicBack (hTRIUS, Germany) and CORFOR V5 (SIDECT, France) provide assistance through elastic bands inserted along the back and posterior side of the thighs. When the wearer of the device flexes the trunk, the elastic becomes constrained, storing energy, making it possible to support part of the back’s efforts. Soft exoskeletons have the advantage of being lightweight, compact, and less expensive, which could facilitate deployment in companies.

### 2.3. Procedure

After a 10-min warm-up of the trunk and lower limb muscles, participants performed isometric maximal voluntary contractions (MVCs) and were familiarized with the experimental tasks.

Participants then performed each of the four different tasks once under each of the five experimental conditions: with the four exoskeletons (“Corfor,” “BionicBack,” “BackX,” and “Laevo”) and without any equipment (“Without exo”). The order of the conditions was randomized. A 5-min familiarization period was planned for each device, during which participants walked, performed stoop and squat flexions/extensions, trunk rotations and inclinations, and performed brief trials of the experimental tasks. All exoskeletons were adjusted to each participant’s morphology, according to the manufacturers’ recommendations. BackX exoskeleton’s assistance was set to the “static mode” for static tasks and to the “dynamic mode” for the lifting tasks. For Laevo FLEX, the medium-stiff gas spring was used.

The four tasks described below were performed in the same order. A 1-min break was given to the subjects between each task and 10 min between each condition.

### 2.4. Experimental tasks

Polishing involves visual inspection and manual correction of the shape and dimensions of aeronautical parts after the milling process. These activities remain predominantly manual due to their high added value, requiring precision, adaptability in gestures, human vision, and decision-making. The improvement of tools and workstations seems to have reached its limits, but back-straining postures persist, and frequent back pain is reported. A comprehensive ergonomic job analysis, conducted by the first author, showed that polishing requires many prolonged or repeated trunk flexions at different angles, as well as occasional load carrying, which makes it a relevant case study. For the experiments, four tasks were selected to represent the diversity of operators’ activities.

#### 2.4.1. Task 1: Interior visual inspection (INSPEC)

**Figure 1.**
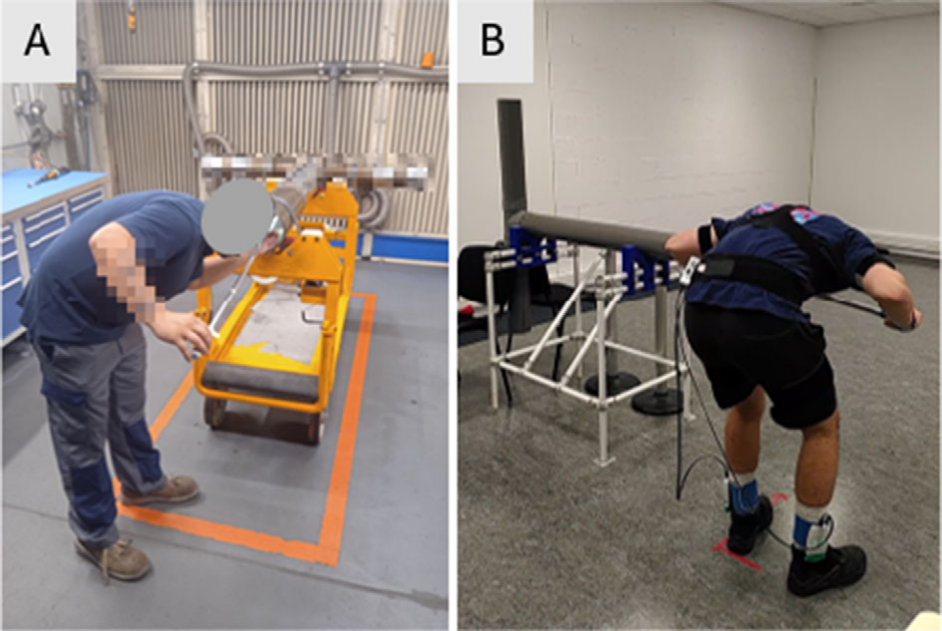
INSPEC task in an ecological situation (A) and in the laboratory setting (B).

Participants had to observe marks inside a tube using a cane fitted with a lamp (156 cm, 500 g), for 40 s. The tube was 93 cm from the ground. Participants performed the task standing with their trunk flexed (knee flexion was free), maintaining their gaze on the marks in the middle of the tube. The cane was not touching the tube (Figure 1).

#### 2.4.2. Task 2: Exterior polishing (POLI)

**Figure 2.**
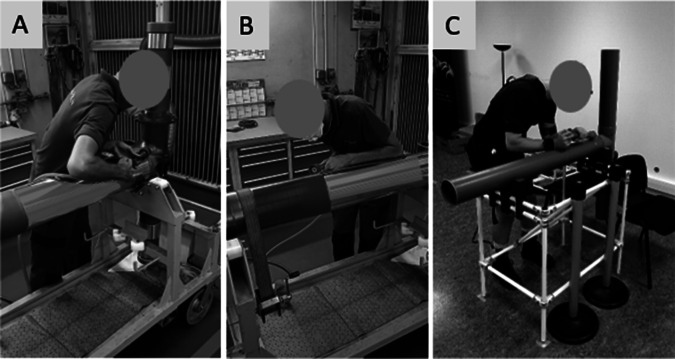
POLI task in ecological situation (A and B) and in the laboratory setting (C).

The subject used a simulated grinder equipped with a force sensor and three indicator lights that illuminated according to the pressure applied (green = low, yellow = medium, and red = high). They were instructed to apply pressure for 3 s on each of the 10 designated stickers placed along the tube – 4 green, 4 yellow, and 2 red – each indicating the required pressure level. Participants were allowed to lean against the tube for support. The task lasted 2 min (Figure 2).

#### 2.4.3. Task 3 and 4: Load carrying 13 (LIFT-13) and 21 kg (LIFT-21)

**Figure 3.**
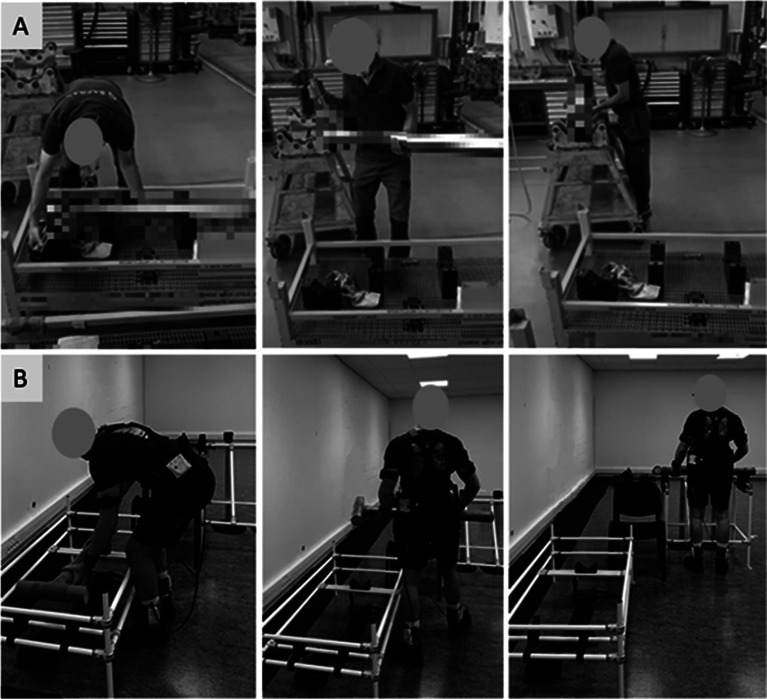
LIFT task in an ecological situation (A) and in the laboratory setting (B).

The LIFT tasks involved carrying an asymmetrical load component of a landing gear. The part measured 110 cm in length, had a diameter of 8 cm, and weighed 13 kg during task 3 and 21 kg during task 4. The subject had to lift the part from a horizontal position in a support located 33 cm above the ground, and place it onto a trolley positioned at a height of 106 cm. This movement required a quarter-turn rotation and a few steps. Subsequently, the participant retrieved the part from the trolley and returned it to the initial support. These actions count as one cycle, and a total of five consecutive cycles were carried out. The lifting technique was unrestricted. The height of the support at the tibia was 38 cm, which limited knee flexion during the tasks (Figure 3).

### 2.5. Data acquisition and analyses

#### 2.5.1. Electromyography

Neuromuscular activity was recorded using the TEA CAPTIV T-SENS surface electromyography (EMG) system (TEA Ergo, Nancy, France). EMG signals were measured for five muscles of the trunk and legs, bilaterally: BF, thoracic ES (TES) at T8 vertebra level, lumbar ES (LES) at L3 vertebra level, obliquus externus (OE), and rectus abdominis (RA). The term ES includes both LES and TES.

Before electrodes were applied, the skin was shaved, scrubbed, and cleaned with alcohol, in accordance with the SENIAM protocol (Hermens and Freriks, 1997). Skin preparation allowed an impedance <5 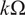 . The EMG signal was sampled at 2,048 Hz. The T-SENS filter stage, which is integrated into the CAPTIV acquisition hardware, is composed of three filters: a Notch Filter (cutoff frequency of 50 Hz), a high pass (second order; cutoff frequency of 16 Hz), and a low-pass filter (second order; cutoff frequency of 725 Hz). The T-SENS does internal root mean square (RMS) computation and transmits results at 128 Hz. The window width for RMS calculation is 78,125 ms.

At the start of the experiment, participants performed two series of isometric MVCs performed against manual resistance, separated by 2 min of recovery. One series included MVCs of all muscles in succession, with at least 30 s of rest between each. For each trial, the RMS value was calculated over successive periods of 100-ms sliding windows using a custom script in MATLAB (R2023a version, The MathWorks Inc., USA). The highest RMS value of each muscle was used as the reference value. For the four tasks, the RMS value of the trial was calculated. RMS values were expressed as a percentage of the reference value (%MVC). The results for the left and right muscles were averaged in the results presented below. To avoid any disturbance of the movement linked to the start or end of the static tasks, the first 5 and last 5 s were removed from the analysis.

#### 2.5.2. Discomfort

**Figure 4.**
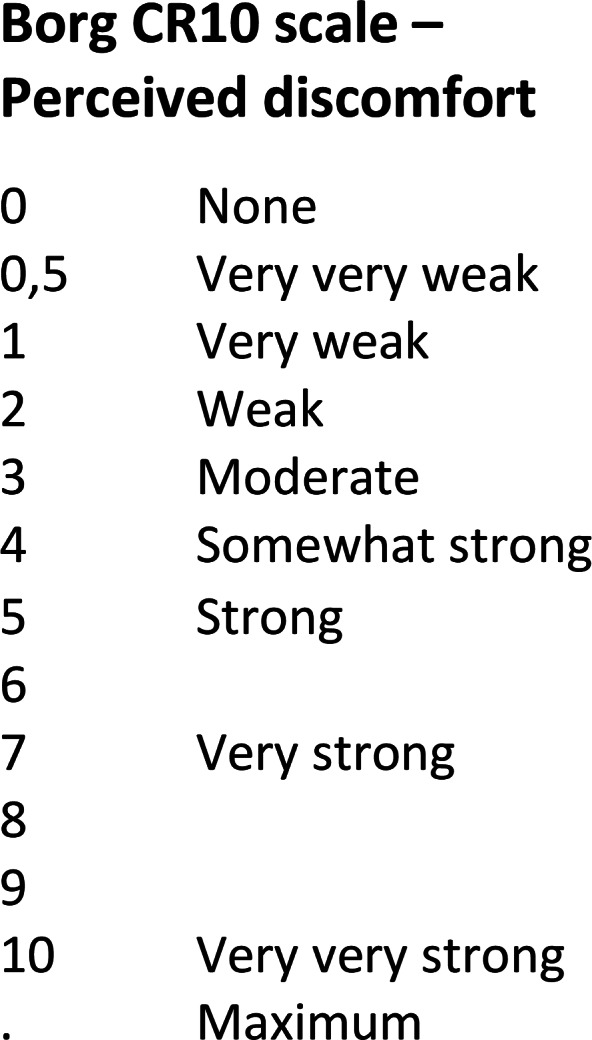
Borg CR10 scale (Borg, 1982).

Perceived discomfort in the lower limbs, torso, and back area was assessed using a Borg CR10 scale (Borg, 1982; Kim et al., 2018; Rashedi et al., 2014; Figure 4). After each task, participants were asked to rate their perceived discomfort in each of these three body areas.

#### 2.5.3. Kinematics

Kinematics analysis was performed using a commercially available inertial measurement unit system (TEA CAPTIV T-SENS Motion, TEA Ergo, Nancy, France). Eleven motion sensors (32 g, 60 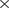 35 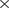 15 mm) were placed in the following configuration: head, upper arms, forearms, sternum, pelvis, thighs, and lower legs. The signals of the sensors were sampled at 128 Hz and synchronized with the EMG data. The system provided measurement accuracy of 2° for yaw/pitch and 0.5° for roll. It recorded triaxial acceleration ( 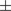 156.96 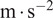 ), angular velocity ( 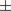 34.9 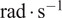 ), magnetic field strength ( 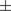 0.25), and spatial orientation. Sensors were placed according to the manufacturer’s guidelines, and the recommended calibration has been performed.

Angular data processing was performed using CAPTIV version L7000 software (TEA, France), which utilizes the Extended Kalman Filter method for signal refinement. Data post-processing was conducted using MATLAB. For the four tasks, the average angle was calculated over the entire acquisition, for the trunk, hips, and knees. For the LIFT tasks, the range of motion (ROM) was also calculated for these same joints. Average angles and ROMs of the right and left knees and hips were averaged bilaterally.

### 2.6. Statistical analysis

Statistical analyses were carried out using JASP software (version 0.16.1.0). Data inspection via the Shapiro–Wilk normality test indicated that the a priori assumption of normality required for repeated-measures analysis of variance (ANOVA) was not consistently met for EMG, kinematics, and perceived discomfort values. Therefore, a Friedman test was used. The significance threshold was set at 5% (*p* < .05). When significant differences were detected, post-hoc pairwise comparison Conover tests were conducted, with *p*-values adjusted using the Bonferroni correction to account for multiple testing. Results are presented as median (interquartile range).

## 3. Results

### 3.1. Electromyography

Abdominal neuromuscular activity, that is, RA and OE muscles, was not significantly impacted by any exoskeleton condition during the four tasks.

**Figure 5.**
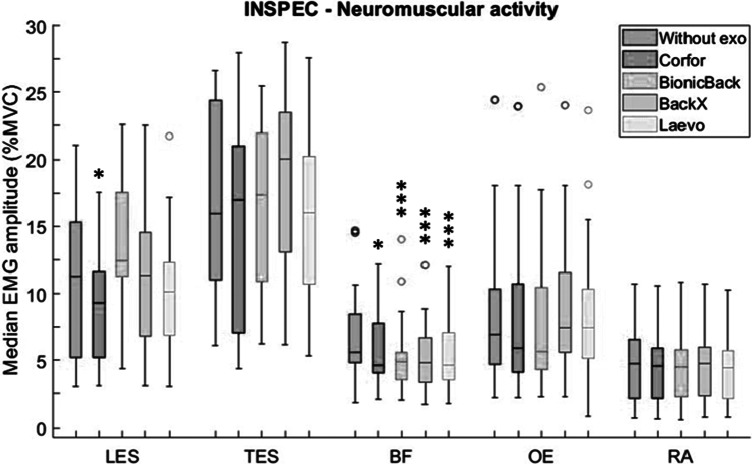
Median EMG amplitude (%MVC) values of lumbar erector spinae (LES), thoracic erector spinae (TES), biceps femoris (BF), obliquus externus (OE), and rectus abdominis (RA), without exoskeleton (blue), with Corfor (red), with BionicBack (yellow), with BackX (purple), and with Laevo (green), for the INSPEC task. In the boxplot, the central mark is the median (horizontal colored bar), the edges of the box are the 25th and 75th percentiles, the whiskers extend to the most extreme data points that are not outliers, and the outliers (o) are plotted individually. Asterisks indicate a significant difference (*p < .05; **p < .01; ***p < .001) with the “Without exo” condition.

The repeated-measures ANOVA revealed a significant main effect of the exoskeleton on LES (*p* < .001), TES (*p* = .019), and BF (*p* < .001) activity during INSPEC. Compared to “Without exo,” Corfor significantly reduced the neuromuscular activity of LES, by 18% (Figure 5). The Conover test also showed a significant difference between BionicBack and Corfor (*p* < .001) and between BionicBack and Laevo (*p* = .014). Compared to “Without exo,” Laevo tended to decrease LES activity by 10% and BionicBack tended to increase it by 11%. The Conover test did not show a significant difference in TES after Bonferroni correction. BF activity was significantly reduced with the four exoskeletons (between 12 and 17% depending on the exoskeleton).

**Figure 6.**
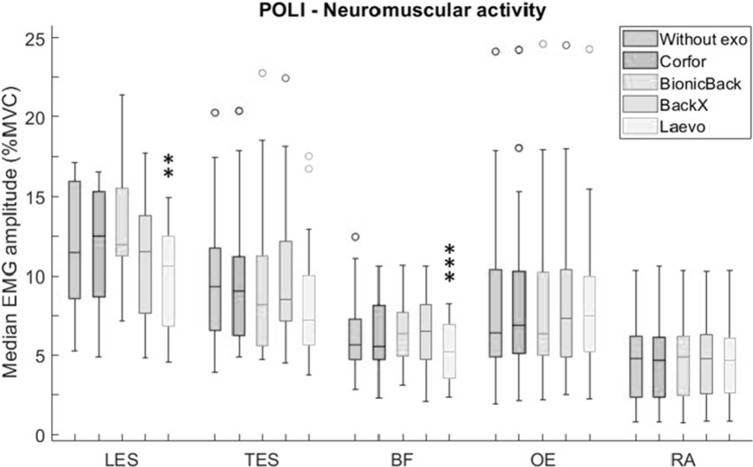
Median EMG amplitude (%MVC) values of lumbar erector spinae (LES), thoracic erector spinae (TES), biceps femoris (BF), obliquus externus (OE), and rectus abdominis (RA), without exoskeleton (blue), with Corfor (red), with BionicBack (yellow), with BackX (purple), and with Laevo (green), for the POLI task. In the boxplot, the central mark is the median (horizontal colored bar), the edges of the box are the 25th and 75th percentiles, the whiskers extend to the most extreme data points that are not outliers, and the outliers (o) are plotted individually. Asterisks indicate a significant difference (*p < .05; **p < .01; ***p < .001) with the “Without exo” condition.

The statistical analysis reported a significant main effect of the exoskeleton on LES (*p* < .001) and BF (*p* < .001) activity during POLI. Only Laevo significantly impacted the neuromuscular activity compared to “Without exo”: 8% reduction on LES and BF (Figure 6). In addition, LES activity with Laevo was significantly lower than with Corfor (*p* = .004) and BionicBack (*p* < .001), and that with BackX was significantly lower than with BionicBack (*p* = .020). BF activity with Laevo was significantly lower than with three other exoskeletons: Corfor (*p* = .032), BionicBack (*p* < .001), and BackX (*p* = .002).

**Figure 7.**
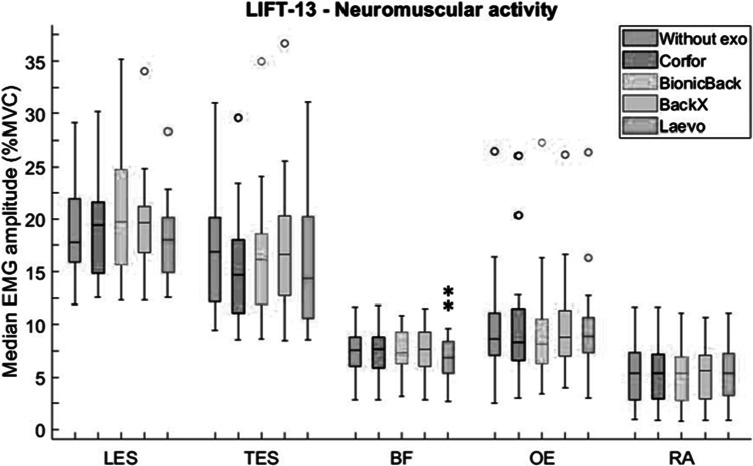
Median EMG amplitude (%MVC) values of lumbar erector spinae (LES), thoracic erector spinae (TES), biceps femoris (BF), obliquus externus (OE), and rectus abdominis (RA), without exoskeleton (blue), with Corfor (red), with BionicBack (yellow), with BackX (purple), and with Laevo (green), for the LIFT-13 task. In the boxplot, the central mark is the median (horizontal colored bar), the edges of the box are the 25th and 75th percentiles, the whiskers extend to the most extreme data points that are not outliers, and the outliers (o) are plotted individually. Asterisks indicate a significant difference (*p < .05; **p < .01; ***p < .001) with the “Without exo” condition.

During LIFT-13, only BF activity was significantly influenced by the exoskeleton (*p* = .004). In detail, its activity was significantly lower by 10% with Laevo compared to “Without exo” (Figure 7). Also, BF activity with Laevo was reduced compared to BionicBack (*p* = .003) and BackX (*p* = .048). None of the four exoskeletons significantly reduced ES activity. A trend of reduced TES activity was observed with Corfor and Laevo of 13 and 15%, respectively.

**Figure 8.**
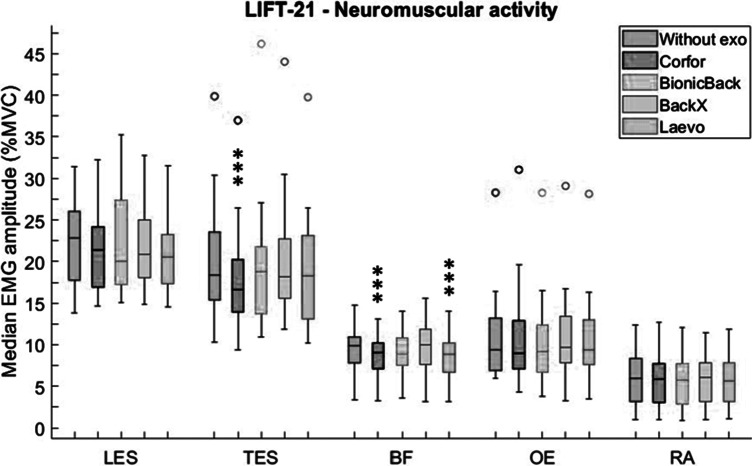
Median EMG amplitude (%MVC) values of lumbar erector spinae (LES), thoracic erector spinae (TES), biceps femoris (BF), obliquus externus (OE), and rectus abdominis (RA), without exoskeleton (blue), with Corfor (red), with BionicBack (yellow), with BackX (purple), and with Laevo (green), for the LIFT-21 task. In the boxplot, the central mark is the median (horizontal colored bar), the edges of the box are the 25th and 75th percentiles, the whiskers extend to the most extreme data points that are not outliers, and the outliers (o) are plotted individually. Asterisks indicate a significant difference (*p < .05; **p < .01; ***p < .001) with the “Without exo” condition.

Finally, during LIFT-21, TES and BF activity were significantly influenced by the exoskeleton (*p* = .002 and *p* < .001, respectively), while the neuromuscular activity of LES was not significantly reduced by exoskeletons, although medians were 7–12% lower across all four conditions (Figure 8). Corfor significantly reduced TES activity by 10%. TES activity was also significantly lower with Corfor compared to BackX (*p* = .008). Compared to “Without exo,” BF activity was significantly lower by 9% with Corfor and by 11% with Laevo. BF activity was also lower in these two conditions compared to BackX (*p* < .001).

### 3.2. Discomfort

**Figure 9.**
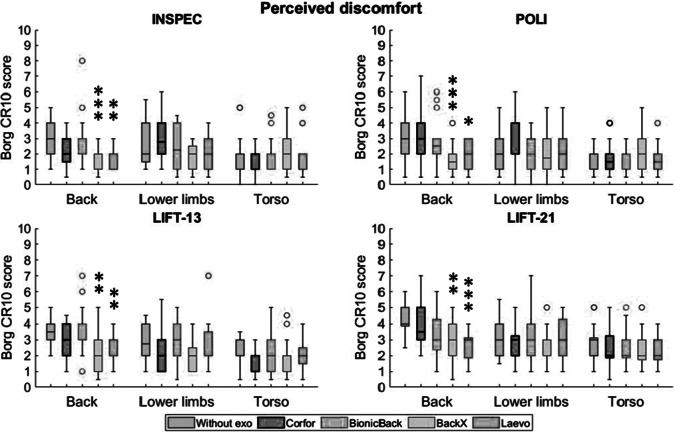
Median discomfort scores (Borg CR10 scale) on back, lower limbs, and torso, without exoskeleton (blue), with Corfor (red), with BionicBack (yellow), with BackX (purple), and with Laevo (green), for the four tasks. In the boxplots, the central mark is the median, the edges of the box are the 25th and 75th percentiles, the whiskers extend to the most extreme data points that are not outliers, and the outliers (o) are plotted individually. Asterisks indicate a significant difference (*p < .05; **p < .01; ***p < .001) with the “Without exo” condition.

The repeated-measures ANOVA revealed a significant effect of the exoskeleton on back perceived discomfort (*p* < .001) during the four tasks. Especially, BackX and Laevo significantly reduced the perceived back discomfort in all four tasks (Figure 9). Median scores decreased from “moderate” to “weak” or “very weak” for the INSPEC and POLI tasks and from “moderate/somewhat strong” to “weak” and from “somewhat strong” to “moderate” for the LIFT-13 and LIFT-21 tasks, respectively. Despite reduced perceived back discomfort with the Corfor (“moderate” vs. “weak” during INSPEC and “moderate/somewhat strong” vs. “moderate” during LIFT-13), these values did not reach the significance level. Perceived back discomfort was significantly lower with BackX compared to BionicBack during INSPEC (*p* < .001) and with BackX compared to Corfor during POLI (*p* = .003). No significant effect was observed on perceived discomfort in the lower limbs and torso.

### 3.3. Kinematics

The statistical analysis reported a significant main effect of the exoskeleton on trunk, hip, and knee kinematics during INSPEC (*p* < .05) and only for trunk and hip kinematics during POLI (*p* < .001). Compared to the “Without exo” condition, Corfor, BackX, and Laevo did not significantly impact the average knee, hip, and trunk angle during the INSPEC and POLI tasks (Table 2). In contrast, BionicBack significantly reduced the trunk flexion angle and increased the hip flexion angle (and knee flexion during INSPEC).

**Table 2.** Median (IQR) of trunk, hip, and knee average angles in flexion

			Without Exo	Corfor	BionicBack	BackX	Laevo
INSPEC	Trunk flexion	Average Angle	66.6	56.2	44.7***	62.0	64.9
			*(15.6)*	*(27.0)*	*(10.1)*	*(15.4)*	*(18.5)*
	Hip flexion	Average Angle	50.5	51.7	71.7***	51.8	38.8
			*(9.7)*	*(15.0)*	*(11.9)*	*(23.0)*	*(28.3)*
	Knee flexion	Average Angle	22.1	25.4	30.4*	21.6	22.3
			*(13.3)*	*(13.4)*	*(24.6)*	*(10.0)*	*(21.1)*
POLI	Trunk flexion	Average Angle	42.3	33.3	28.5***	36.5	39.9
			*(23.7)*	*(18.1)*	*(15.7)*	*(18.5)*	*(20.1)*
	Hip flexion	Average Angle	14.5	13.3	27.4***	20.0	20.1
			*(15.1)*	*(14.8)*	*(19.4)*	*(19.5)*	*(9.3)*
	Knee flexion	Average Angle	8.7	10.5	8.6	9.7	10.8
			*(9.7)*	*(7.4)*	*(10.5)*	*(6.7)*	*(15.4)*

*Note.* Asterisks indicate a significant difference (*p < .05; **p < .01; ***p < .001) with the “Without exo” condition.

There was also a significant main effect of the exoskeleton on trunk, hip, and knee kinematics during the lifting tasks (*p* < .05). The median of the average trunk angle in both LIFT tasks was lower with all four exoskeletons. The reduction was significant for Corfor, BionicBack, and BackX in LIFT-21 (Table 3). In both LIFT tasks, BionicBack significantly reduced the average trunk angle and ROM, and increased the average hip angle.

**Table 3.** Median (IQR) of trunk, hip, and knee ROM and average angle

			Without Exo	Corfor	BionicBack	BackX	Laevo
LIFT–13	Trunk flexion	ROM	59.5	51.7	38.1**	64.2	58.2
			*(21.8)*	*(23.8)*	*(24.2)*	*(21.2)*	*(11.6)*
		Average angle	32.9	21.7	18.0***	23.7	25.5
			*(16.8)*	*(12.2)*	*(14.0)*	*(13.9)*	*(9.0)*
	Hip flexion	ROM	97.6	95.4	102.0	86.9	85.2*
			*(19.5)*	*(18.6)*	*(13.7)*	*(22.4)*	*(16.5)*
		Average angle	21.3	23.9	30.5***	22.6	22.5
			*(12.1)*	*(12.3)*	*(4.9)*	*(13.1)*	*(8.8)*
	Knee flexion	ROM	68.8	67.4	70.0	62.2	68.5
			*(15.9)*	*(13.7)*	*(15.6)*	*(8.5)*	*(11.4)*
		Average angle	17.7	17.4	18.6	18.3	19.9
			*(5.1)*	*(5.2)*	*(8.9)*	*(7.5)*	*(7.8)*
LIFT–21	Trunk flexion	ROM	59.1	49.1	42.6*	62.5	61.2
			*(24.3)*	*(27.4)*	*(17.5)*	*(24.6)*	*(22.7)*
		Average angle	35.8	22.8**	18.3***	25.7*	28.3
			*(16.2)*	*(16.2)*	*(15.3)*	*(15.8)*	*(16.0)*
	Hip flexion	ROM	94.7	95.9	104.4	85.6	89.9
			*(16.1)*	*(18.3)*	*(11.5)*	*(21.7)*	*(25.8)*
		Average angle	21.4	26.4	32.5**	22.2	23.7
			*(9.7)*	*(12.2)*	*(8.5)*	*(9.8)*	*(9.8)*
	Knee flexion	ROM	69.0	65.5	70.2	62.3**	63.1
			*(11.1)*	*(13.7)*	*(13.9)*	*(10.0)*	*(11.2)*
		Average angle	18.5	17.5	17.7	19.2	18.9
			*(6.5)*	*(7.7)*	*(8.2)*	*(6.8)*	*(9.5)*

*Note.* Asterisks indicate a significant difference (*p < .05; **p < .01; ***p < .001) with the “Without exo” condition.

## 4. Discussion

The present study investigated the biomechanical effects of four different passive back exoskeletons during simulated tasks specific to aeronautics. The main findings were that (i) Laevo and Corfor provided the most reduction in ES neuromuscular activity, (ii) Laevo and BackX reduced significantly back discomfort, and (iii) Laevo, Corfor, and BackX did not cause consistent adverse effects (muscle overuse, changes in kinematics, and increased discomfort).

### 4.1. Beneficial effects

#### 4.1.1. Reduction in neuromuscular activity

Laevo and Corfor reduced ES neuromuscular activity to some extent. Indeed, Laevo significantly reduced LES activity by 8% during the POLI task, and Corfor yielded a significant 18% reduction in LES during INSPEC and a significant 10% reduction in TES during LIFT-21.

This ES muscle relief is in line with the literature, showing varying reductions from 6 to 61% and often considered as a sign of reduced workers’ exposure to physical risk factors related to LBP (De Looze et al., 2016; Theurel and Desbrosses, 2019; Kermavnar et al., 2021). However, contrary to expectations, there were rather few significant effects of exoskeletons on the ES during INSPEC. Other studies, evaluating anterior versions of the Laevo exoskeleton during static trunk bending tasks, reported 11–57% significant reductions in ES activity (Bosch et al., 2016). The use of BackX has also demonstrated a reduction in back neuromuscular activity by up to 47% (Madinei et al., 2020b). However, these studies involved lower trunk flexion angles, with values of ~40° or even less. Given the substantial trunk flexion angle ( 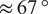 ) induced during INSPEC, the lack of significant reduction in LES in the present study could be explained by the flexion-relaxation phenomenon, commonly observed at the end of trunk flexion (Toussaint et al., 1995). This phenomenon involves a sudden reduction or silence of the myoelectric activity of the LES during full trunk flexion, occurring at varying lumbar flexion angles depending on the individual. Due to the spinal extensors’ relaxation, the flexion torque is then supported by the ligaments of the spine (Koopman et al., 2019; Gouteron et al., 2022). For instance, in the present study, 5 out of 19 participants had a mean EMG amplitude of LES below 5% MVC during this task. Therefore, a reduction in EMG did not necessarily imply that the load applied to the lower back was reduced. This underscores the possible limitation of using average neuromuscular activity measurements for this type of task. Consistent with this, it has been shown that peak compressive and shear forces, as well as peak ES muscle force, can be reduced by back exoskeletons even in the absence of significant changes in average ES neuromuscular activity (Favennec et al., 2024). Therefore, the absence of significant EMG differences does not rule out a potential unloading effect of the exoskeleton on passive or deep musculoskeletal structures. Another result is consistent with the flexion-relaxation hypothesis: the increase in muscle activity in the LES with the BionicBack during INSPEC could be explained by the significantly lower back flexion in this condition (median value of only 45° wrt. 67° without exoskeleton). In the BionicBack condition, some participants were no longer subject to the flexion-relaxation when they performed the task, leading to an increase in the muscle activity of the back compared with the other conditions.

Despite the effect of the Corfor on TES during LIFT 21, exoskeleton support does not appear to be sufficient to significantly reduce the neuromuscular activity of ES during lifting tasks. Other studies report more significant results on this type of task, with a reduction of ES activity by 12–24% with rigid exoskeletons (Poon et al., 2019; Alemi et al., 2020; Madinei et al., 2020a; Refai et al., 2024) and by 7–16% with soft ones (Lamers et al., 2018; Schwartz et al., 2021). Besides the differences related to the model or version of exoskeletons used in various studies, this difference could be explained mainly by the difference in experimental tasks. Indeed, most laboratory protocols typically include simple, highly controlled lifting/lowering tasks. More complex tasks like those in the present study (lifting/lowering at different heights, rotation, and a few steps) could marginalize the effects of exoskeletons on segments of the task. For instance, Luger et al. (2021), who studied a relatively complex lattice box lifting task, also did not observe any reduction in muscle activity. Similarly, Reimeir et al. (2023) observed significant reductions only by segmenting their complex load-carrying task. In addition, Schwartz et al. (2023) did not observe any significant reduction with the BackX during a dynamic lifting task with a 15 kg load. Similar to the present study, the relatively high load compared to other studies (Alemi et al. (2020) 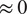 and 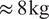 ; Madinei et al. (2020a): 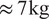 ) may explain the limited assistance provided by the exoskeleton. Indeed, as previous research suggests, increasing the load handled could reduce the benefits provided by passive systems (Abdoli-E et al., 2006).

In addition, BF neuromuscular activity was significantly lower in the four tasks when using the Laevo, with reductions ranging from 8 to 17%. Other studies observed comparable results with the Laevo use, with 8–36% reduction depending on the study (Bosch et al., 2016; Luger et al., 2021; Schwartz et al., 2023). Wearing the CORFOR yielded similar effects only for INSPEC and LIFT-21. In addition, BF activity was significantly reduced only for INSPEC with BionicBack and BackX. This shows that the exoskeleton takes over part of the hip extensor moment, which can be interpreted as an advantage of exoskeleton usage in forward bending work (Bosch et al., 2016). However, the low BF’s demand in %MVC should be considered and would probably mitigate the possible benefit associated with the reduced BF recruitment ( 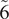 %MVC for static tasks and 7–10%MVC for LIFT in the present study).

These results show that in terms of reduction of neuromuscular activity, the observed effects do not appear to be solely specific to the rigid or soft design of the exoskeleton. The variability of exoskeleton effects across tasks and models highlights the need to assess different exoskeletons across various trunk-bending tasks that reflect real work conditions to accurately assess their potential benefits before implementation in the workplace.

#### 4.1.2. Reduction in perceived back discomfort

The BackX and Laevo exoskeletons significantly reduced the perceived back discomfort in all four tasks. This is consistent with previous studies showing a significant decrease in perceived discomfort in the lower back with rigid exoskeletons, including earlier versions of the Laevo and BackX (Bosch et al., 2016; Baltrusch et al., 2018; Alemi et al., 2020). Corfor slightly reduced discomfort during the INSPEC and LIFT tasks, but not enough to be significant. Reductions in perceived back discomfort, mainly with rigid exoskeletons, show that they could provide relief to operators during these different polishing tasks, thereby improving well-being at work and potentially reducing the risk of LBP. Indeed, the intensity of perceived musculoskeletal discomfort has been recognized as a risk factor for the occurrence of LBP (Hamberg-van Reenen et al., 2008). Discomfort reductions also support the back muscles’ relief seen previously with Laevo, and to a lesser extent with Corfor. However, the decreased back perceived discomfort with BackX was not associated with such a back-muscle activity reduction, underlying discrepancies between perceived parameters and objective parameters, which effects might be difficult to tease out if their amplitude is limited. However, BionicBack did not reduce discomfort. It is likely that the rigid part in the back, which was reported as uncomfortable, attenuated any possible back relief due to the support of the exoskeleton.

### 4.2. Adverse effects

The observed reductions in EMG amplitude, particularly with Laevo and Corfor, were not attributable to kinematic changes and, therefore, reflect reductions in lumbar muscle strain. Indeed, only occasional modifications were observed: reduction of hip ROM with Laevo during LIFT-13 and reduction of the trunk flexion angle with Corfor during LIFT-21, without involving any other significant modification. BackX also did not modify kinematics, but as mentioned earlier, this device did not influence ES activity.

Only BionicBack significantly impacted the participants’ kinematics in all four tasks, reducing average and ROM trunk flexion. This is most likely due to the rigid part of the exoskeleton in the back, which limited lumbar flexion. This was considered restrictive and uncomfortable by several participants (a higher back discomfort score of 2 points was observed by four participants with the BionicBack). To compensate for this limitation in trunk flexion, participants flexed their knees and hips more.

Rigid exoskeletons have been suggested to induce a hyperextension of the knees, due to the force applied to the front of the thighs (Bosch et al., 2016; Luger et al., 2021). These authors hypothesized that it could lead to increased BF activity as a compensatory mechanism, potentially posing risks to the users’ backs and/or knees. However, the present study did not confirm this hypothesis, as neither knee ROM nor average flexion showed hyperextension, and BF activity did not show a significant muscle increase in any of the tasks.

The abdominal muscles (RA and OE) were not impacted by the four exoskeletons in any of the tasks. The lack of overexertion of the abdominal muscles indicates that participants did not need to force against the exoskeleton during trunk flexion, as reported in other studies (Alemi et al., 2019; Baltrusch et al., 2019; Refai et al., 2024). Perceived discomfort in the lower limbs and torso was not impacted by any of the four exoskeletons, in any of the tasks. The Laevo FLEX vest, transferring force to the torso, seems more comfortable than the rigid chest pad of earlier versions, which had increased torso discomfort (Bosch et al., 2016; Amandels et al., 2019; Madinei et al., 2020a). Therefore, the four tested exoskeletons did not significantly have negative impacts on the abdominal muscles or other body regions, where points of assistance transfer of the exoskeleton are located.

### 4.3. Limits

This study has certain limitations that should be acknowledged. First, using the average EMG amplitude over the entire task may obscure specific effects of exoskeletons during different phases of dynamic tasks. However, this approach was considered appropriate, as effects that are not clearly detectable over the full task duration are unlikely to have a substantial impact on neuromuscular strain. Second, the participants had only a very short period to familiarize themselves with the exoskeleton. This implies a possible suboptimal use of the device, even if these devices were easy to use. Favennec et al. (2024) showed that biomechanical variables, such as thoracic kinematics or contact pressure perception, require three to four periods of 1-hr familiarization sessions to stabilize for a soft-back exoskeleton.

Another limit is that the participant sample consisted of relatively young and physically active men, which does not fully represent the broader working population. Caution is, therefore, warranted when generalizing these results to female, older, injured, and/or overweight workers.

Furthermore, the absence of adverse effects of exoskeletons during this study does not imply that none would emerge in real-world conditions. While the tasks closely resemble actual work, the short duration of the trials compared to field conditions limits their applicability. Discomfort or kinematic changes may develop over prolonged use. Therefore, in-field testing is crucial, particularly for polishing tasks and more broadly for all activities involving trunk bending.

## 5. Conclusion

The Laevo FLEX exoskeleton was the only one to significantly reduce both neuromuscular activity and perceived back discomfort without causing adverse effects. The CORFOR exoskeleton also led to significant reductions in neuromuscular activity, while the BackX exoskeleton notably decreased perceived back discomfort. The Laevo FLEX exoskeleton appears particularly beneficial when polishing in the aeronautical field, which contains many static trunk bending tasks and occasional load carrying. Future research in real-world settings, incorporating worker/activity-centered assessments and acceptance-related factors, is needed to confirm its effectiveness. Moreover, the effect of back exoskeletons during intense flexion tasks requires further investigation, as neuromuscular activity alone may not fully capture back strain in such conditions.

## Data Availability

Data availability is not applicable to this article as no new data were created or analyzed in this study.
